# Molecular mechanisms of suxiao jiuxin pills in ameliorating post-acute myocardial infarction inflammatory response: a combined network pharmacology, Mendelian randomization, and experimental validation study

**DOI:** 10.3389/fcvm.2026.1852925

**Published:** 2026-07-16

**Authors:** Yugen Shi, Wenjing Yi, Xue Feng, Qingshan Zhang, Shuai Bao, Li Sun, Suhua Yan, Nannan Li, Xiaolu Li

**Affiliations:** 1 Shandong University of Traditional Chinese Medicine, Jinan, Shandong, China; 2 Department of Cardiology, The First Affiliated Hospital of Shandong First Medical University & Shandong Provincial Qianfoshan Hospital, Shandong Medicine and Health Key Laboratory of Cardiac Electrophysiology and Arrhythmia, Jinan, Shandong, China; 3 Department of Emergency Medicine, The First Affiliated Hospital of Shandong First Medical University & Shandong Provincial Qianfoshan Hospital, Jinan, Shandong, China; 4 Department of Chinese Medicine Ophthalmology, The First Affiliated Hospital of Shandong First Medical University & Shandong Provincial Qianfoshan Hospital, Jinan, Shandong, China

**Keywords:** acute myocardial infarction, fos, machine learning algorithms, NAMPT, suxiao jiuxin pill

## Abstract

**Purpose:**

In recent years, Suxiao Jiuxin Pill (SJP) has emerged as a potential treatment for various cardiovascular diseases, the exact molecular mechanisms remain poorly characterized. Consequently, this study seeks to investigate the target genes associated with SJP's active components in AMI, as well as the underlying biological processes, utilizing network pharmacology (NP) and Mendelian randomization (MR) analysis.

**Methods:**

To unravel SJP's targets and its regulatory mechanisms against AMI, we combined NP, MR, and molecular docking strategies. A rat MI model was established by ligating the LAD coronary artery at the designated site. PCR and immunofluorescent labeling were applied to ovserve the expression of NAMPT and FOS.

**Results:**

Totally 44 DE-TGs were gained by intersecting 689 DEGs and 969 predicted target genes. Next, two key target genes, NAMPT and FOS, showing markedly upregulated expression in AMI samples. Observations showed that these genes were co-enriched in the “Leishmania Infection” and “Chemokine Signaling Pathway”. Moreover, these key target genes showed robust associations with various immune cells, of which NAMPT exhibited a strong positive correlation with neutrophils (co*r* = 0.65). Molecular docking revealed NAMPT bound to oleic acid (−5.9 kcal/mol) and FOS bound to pentadecanol (−5.4 kcal/mol). The PCR and immunofluorescence assay results showed that FOS was consistent with the predicted results. Echocardiographic assessments and inflammatory factor expression analyses confirmed that SJP ameliorated cardiac function and alleviated the inflammatory response in MI rats.

**Conclusion:**

This work further delivers a fresh conceptual framework for deciphering the mechanistic basis of SJP's clinical utility in AMI management.

## 1 Introduction

Globally, acute myocardial infarction (AMI) poses a grave threat to health as a common cardiovascular disease. This condition carries the risk of precipitating lethal outcomes, including malignant arrhythmia, cardiogenic shock, and sudden cardiac death (1, 2). The pathogenesis primarily involves unstable plaque rupture with subsequent thrombus formation, leading to coronary artery occlusion, myocardial ischemia and hypoxia, and ultimately myocardial necrosis (3). Although PCI represents an established clinical strategy that mitigates the incidence of acute mortality and ventricular remodeling (4) and contributes to improved AMI prognosis (5–9), acute myocardial infarction persists in ranking among the foremost causes of death globally (10). Supporting this, a study of 19,781 individuals with coronary artery disease reported a 23.3% prevalence of MI (11), thereby creating a critical demand for novel therapeutic development. and elucidate the underlying mechanisms for the prevention and treatment of acute myocardial infarction.

In recent years, traditional Chinese medicine has received increased research attention. Traditional Chinese Medicine has a long history of over two thousand years and is becoming increasingly popular as a complementary therapy worldwide (12). At present, the treatment principle of Western medicine for patients with acute myocardial infarction is ischemia-reperfusion, and there are also many drugs used to treat AMI. Current drug therapies, despite their progress, continue to pose significant challenges including side effects and adverse reactions (13). In this context, Suxiao Jiuxin Pill (SJP), a classic Chinese patent medicine based on Chuanxiong [Ligusticum chuanxiong, Pharmacop of China (2005)] and Borneol [BingPian, *Pharmacop of China (2005)*], has been applied in managing coronary heart disease and related ischemic events (14). Its traditional mechanism, as described in Chinese medicine theory, involves promoting circulation, eliminating stasis, and providing analgesia (15). Clinical research supports its role in combating atherosclerosis; specifically, ligustrazine—an active component of SJP—modulates key lipid factors by reducing LDL and increasing HDL/HDL2 levels, which contributes to plaque inhibition and lesion regression (16) Moreover, multiple *in vivo* studies confirm that SJP enhances hemodynamics, curbs platelet aggregation, and limits thrombus formation in arteries. Research shows that SJP can reduce the inflammatory reaction in atherosclerosis (17). For decades, it has been widely used in clinical treatment and self medication therapy. However, research on the specific mechanism by which it improves acute myocardial infarction is limited.

Traditional Chinese medicine prescriptions generally consist of two or more kinds of Chinese medicine flavors. Due to the complexity of patients’ conditions and the diversity of pathogenesis, it is difficult for a single drug to meet the needs of patients with changeable and complex conditions (18). TCM compounds are characterized by multi-target and multi-pathway mechanisms of action (19), necessitating the use of modern analytical approaches to elucidate their active components and therapeutic mechanisms. An interdisciplinary approach, network pharmacology facilitates the identification of novel therapeutic targets and underlying mechanisms by deciphering the molecular basis of drug actions across diseases through multilayered interaction networks of genes, targets, and pathways (20–22). Its integrality, systematicness, and comprehensiveness are very similar to those of traditional Chinese medicine, so it is very suitable for the study of pharmacological mechanism of traditional Chinese medicine (23–25). This method provides important information and scientific reference for the discovery of TCM treatments (26). Additionally, By harnessing genetic variants as instrumental variables, Mendelian randomization (MR) serves as a genetics-driven epidemiological method for robust causal inference regarding exposure-outcome relationships (27, 28). This technique circumvents inherent limitations of observational studies, such as confounding and reverse causality, thereby serving as a robust complement to conventional experimental designs (29).

In this study, AMI-related transcriptome sequencing data from public databases and network pharmacology were used to comprehensively explore the potential mechanism of SJPs on myocardial infarction. The main therapeutic targets were identified by Mendelian randomization analysis. Furthermore, *in vivo* experiments were performed to validate the key targets and therapeutic effects of SJP in myocardial infarction. Our findings aim to advance the understanding of SJP's molecular mechanisms in treating AMI and provide scientific evidence for its clinical application.

## 2 Material and methods

### 2.1 Data source

The GSE66360 and GSE61144 datasets were downloaded from the Gene Expression Omnibus (GEO) database. The whole blood samples from 49 AMI and 50 control patients in the GSE66360 dataset (training cohort) (30), as well as whole blood samples from 7 AMI and 10 control patients in the GSE61144 dataset (validation cohort) (31).

Active components and their corresponding target genes of SJP in the BATMAN-TCM database were identified via searches using “ChuanXiong” and “BingPian”. All plant names used in this study have been verified against the Medicinal Plant Names Services (MPNS; http://mpns.kew.org), with an access date of December 9, 2025. The botanical names of all plants involved in this study underwent validation against the Medicinal Plant Names Services (MPNS). The potential active components and their corresponding target genes were retrieved and filtered based on a Score cutoff of ≧ 20, with the genes of the active components meeting the screening criteria selected as the predicted target genes for subsequent analysis (Supplementary Data Sheet 1).

### 2.2 Identification of DEGs

Through the limma (v 3.54.0) (32), DEGs were derived from 2 groups of the training cohort [|log_2_Fold Change (FC)| > 1 and adj.*P* < 0.05]. Visualization of DEGs result was achieved using the ggpubr (v 0.6.0) (33) and ComplexHeatmap (v 2.14.0) (34), respectively. Moreover, with the aim of observing the interactions between drugs and active components, an interaction network involving ChuanXiong, BingPian, and their active components was rendered using Cytoscape (v 3.9.1) (35).

### 2.3 Identification and enrichment analysis of differentially expressed target genes (DE-TGs)

So as to obtain DEGs related to target genes linked to SJP in the treatment of AMI, the overlap of DEGs and predicted target genes was generated via the VennDiagram (v 1.7.3) (36), resulting in the detection of DE-TGs in AMI. Furthermore, enrichment analyses were performed via ClusterProfiler (v 4.7.1.3) (37) and org.Hs.eg.db (v 3.16.0) (38). The GO encompassed BP, CC, and MF. These findings were graphically illustrated with the enrichplot (v 1.18.0) (39).

### 2.4 Screening of instrumental variables (IVs)

The data for ‘DE-TGs’ from the IEU Open GWAS database were used as exposure factors. Correspondingly, the AMI dataset (ukb-a-533) was specified as the outcome variable for subsequent analyses. This dataset comprised 10,894,596 SNPs from 337,199 samples, with case = 3,927 and control = 333,272. MR studies rely on three basic assumptions: (1) IVs have a strong and significant correlation with exposure; (2) IVs are uncorrelated with confounding factors; (3) the impact of IVs on outcomes is exclusively mediated by exposure, with no other channels involved. At the start, we deployed the extract instruments function from the Two Sample MR (v 0.5.8) (40) to pull exposure factors and select IVs that correlated significantly with these factors (*P* < 5*10–8). Thereafter, we implemented the parameter settings of r2 = 0.001, kb = 20 and clump = TRUE to exclude LD-positive IVs from our study. We also calculated the F-statistic (F = beta2/se2) and deemed IVs to be suitably robust when F > 10. Besides, we carried out outcome variable extraction using the extract outcome data function, filtering them alongside IV exposure factors (proxies = TRUE; rsq=0.8).

### 2.5 Detection of genes with causal links to AMI

Primarily, the harmony_data function was applied to align effect sizes and ensure consistency across datasets. Afterwards, the mr function, combined with five algorithms [MR-Egger (41), Weighted median (42), Inverse variance weighted (IVW) (43), Simple mode (40), and Weighted mode (44)], was used to perform MR analyses. Leveraging the superior performance of the IVW method in causal association detection, we identified a significant causal relationship between DE-TGs and AMI. Importantly, An OR exceeding 1 signaled that exposure factors acted as risk contributors, while an OR below 1 implied these factors functioned as protective mediators.

In addition, a sensitivity analysis was conducted. First, heterogeneity was examined using the mr_heterogeneity function (*P* > 0.05). This was followed by the performance of a horizontal pleiotropy test (45) was implemented to identify confounding factors using the mr_pleiotropy test (*P* > 0.05). Subsequently, the LOO sensitivity assessment (46) was conducted by gradually removing each SNP to evaluate how excluding individual SNPs affected the overall findings.

### 2.6 Identification of key target genes

Based on the genes flagged as causally linked to AMI through MR analysis, we constructed a SVM-RFE algorithm and LASSO Cox regression analysis to further pinpoint candidate key target genes using the caret (v 6.0–93) (47) and glmnet (v 4.1.4) (48), respectively. In conclusion, the candidate key target genes were gained by overlapping the results of both machine learning via the VennDiagram. Thereafter, we employed ggplot2 (v 3.4.1) (49) to illustrate the expression patterns of these candidate core target genes in AMI and control specimens across the two cohorts. Critically, the genes showing consistent expression trends with significant differences in two cohorts were deemed as key target genes. And we also displayed the chromosomal distribution of key target genes via Circos.

### 2.7 Function of key target genes

With the intention of gaining insights into key target genes related biological functions and pathways, Gene Set Enrichment Analysis (GSEA) was performed through ClusterProfiler. The C2: KEGG gene set was obtained from the MSigDB database.

### 2.8 Immune infiltration analysis

In the first place, samples with no notable variation in the content of 22 immune cells (50) were excluded from the training cohort (*P* > 0.05). Subsequently, the proportions of these cells between 2 groups were determined and compared using the CIBERSORT algorithm (v 1.03) (51). Subsequently, the correlations existing among differential immune cells, in addition to their relationships with key target genes were explore.

### 2.9 Network and molecular docking analysis

Correlations were assessed via Inference scores using the CTD database. For the prediction of TFs that modulate key target genes, we leveraged the JASPAR database and further generated a visualized TF-mRNA regulatory network. Additionally, the 3D structures of drugs were downloaded from PubChem, whereas the structural models of core proteins were acquired through the PDB database. Next, AutoDock Vina (52) software was applied to obtain binding energies.

### 2.10 Animals and protocols

SJP was is mainly composed of Chuanxiong and BingPian, but its formula is still in the confidential stage，and was sourced from commercial supplies produced by the Tianjin Pharmaceutical Da Ren Tang Group Co.Ltd. NO.6 Traditional Chinese Medicine Factory (batch number: 912203). Male Sprague-Dawley rats aged 8 weeks were sourced from Beijing Vitalriver Company and kept in a standard SPF animal housing facility. All procedures complied with the National Institutes of Health Guide for the Care and Use of Laboratory Animals (No. 85–23, revised 1996), received approval from the Institutional Animal Care and Use Committee and was approved by the Ethics Committee of the First Affiliated Hospital of Shandong First Medical University (2024121501). All experiments were conducted after the rats had undergone environmental adaptation.

The animals were randomly divided into 4 groups (*n* = 20 each): Sham group, Sham group with SJPs pretreatment (Sham + SJP), AMI group (MI), AMI group with SJPs pretreatment (MI + SJP). According to the grouping, all rats were given SJPs (a dose of 900 mg/kg/d once daily) (53) or an equal volume of physiological saline by gavage treatment 7 days before surgery.

#### 2.10.1 MI surgery

This animal experiment was performed in compliance with the ARRIVE guidelines, the Animals (Scientific Procedures) Act 1986 (and its associated protocols), and EU Directive 2010/63 (regulating the welfare of laboratory animals in scientific research). After a 7-day treatment regimen, rats were anesthetized with 3% pentobarbital sodium (30 mg/kg), followed by tracheotomy, endotracheal intubation, and connection to a small animal ventilator for respiratory support. A 3-cm incision was made in the left thoracic wall through the fourth left intercostal space, and the heart was exposed via pericardiotomy. As reported in previous studies, MI was induced by ligating the left anterior descending (LAD) coronary artery with a 6–0 silk suture—2 mm from its origin, at the junction between the pulmonary conus and left atrium. Electrocardiographic changes were monitored using the BL-420S Animal Biological Function Detection System (Taimeng, China), with MI confirmed by ST-segment elevation and left ventricular stiffness. For the sham-operated group, only thoracotomy and pericardiotomy were performed without arterial ligation. Postoperatively, rats were allowed to regain consciousness from anesthesia, maintained at a constant body temperature of 37 °C on a heating pad, and then returned to their individual cages. Subsequent to surgery, the animals received a further 7-day course of continuous intragastric medication.

Assessment of cardiac function, Immunofluorescence, PCR, and inflammatory markers was performed 7 days after ligation.

#### 2.10.2 Quantitative real-time PCR (RT-PCR) analysis

Total RNA was isolated using RNA-easy™ Isolation Reagent (Vazyme Biotech Co.), with complementary DNA (cDNA) subsequently synthesized via reverse transcription employing the PrimeScript™ RT kit (Vazyme Biotech Co.). Relative mRNA expression levels were quantified on a Bio-Rad iQ5 Multicolor RT-PCR System (Bio-Rad Laboratories, Hercules, CA, USA). Target gene expression was normalized to GAPDH levels and calculated using the 2^−*ΔΔ*Ct^ method. Primer sequences were designed as follows:

Fos-Forward: 5′-CAAACCGACCTACTGTCCC -3′; Fos-Reverse: 5′- ACCAACAACCTTGTCGTCATAT -3′;
NAMPT-Forward: 5′-ATGCCGTGAAAAGAAGACAG -3′; NAMPT-Reverse: 5′-TCCAGTTGGTGAGCCAGTAG -3’;
GAPDH-Forward: 5′-TCTCTGCTCCCTGTTCT -3′; GAPDH- Reverse: 5′-ATCCGTTCACACCGACCTTC -3′.

#### 2.10.3 Assessment of cardiac function

Transthoracic echocardiography was performed at 7 days post-ligation to assess cardiac function. Rats were examined under anesthesia; M-mode recordings obtained with a 30 MHz transducer were used to quantify left ventricular (LV) internal diameter in the short-axis view. Left ventricular ejection fraction (LVEF) and left ventricular fractional shortening (LVFS) were subsequently analyzed with the Vevo 2000 workstation.

#### 2.10.4 Masson's trichrome staining

Rat cardiac tissues were harvested, fixed, paraffin-embedded, and sectioned into 5-*μ*m thick slices. Cardiac fibrosis across experimental groups was quantified using a Masson's trichrome staining kit (cat. no. HT15, Sigma-Aldrich, St. Louis, MO, USA) in strict accordance with the manufacturer's instructions. The infarct area percentage was calculated using the formula: (fibrotic area/total left ventricular area) × 100%.

#### 2.10.5 Immunofluorescent staining

Harvested hearts were processed into FFPE tissue blocks for HE staining or conventional immunohistochemical analysis. Tissue sections were incubated with 3% hydrogen peroxide for 30 min and then rinsed three times with PBS. Following blocking with 5% BSA for 30 min, the slides were incubated overnight at 4 °C with primary antibodies against Fos (ab289723, Abcam) and NAMPT (CQA8218, Cohesion Biosciences). For negative control groups, primary antibodies were replaced with PBS. Subsequently, the sections were incubated with species-compatible secondary antibodies for 1 h at room temperature, followed by counterstaining with DAPI prior to mounting.

#### 2.10.6 ELISA

IL-1, TNF-α, NE, HIS, and cTNI were detected in blood and heart tissue samples on the 7th day after MI. Blood samples were collected into Eppendorf tubes preloaded with 30 µL of 10% EDTA-Na₂ (1 mg/mL, Sigma-Aldrich) and 40 µL of aprotinin (500 kIU/mL, Sigma-Aldrich). Following centrifugation, supernatants were harvested promptly and preserved at −80 °C. A commercial ELISA kit (Reed Biotech, Wuhan, China) was used to determine target concentrations.

### 2.11 Statistical analysis

All statistical computations were conducted using SPSS 17.0. Quantitative data are reported as mean ± standard error. Group comparisons were carried out by t-test or analysis of variance, followed by the minimum significance difference *post-hoc* test; significance was determined at the *P* < 0.05 threshold.

## 3 Results

### 3.1 A sum of 689 DEGs was acquired

**Figure 1:**
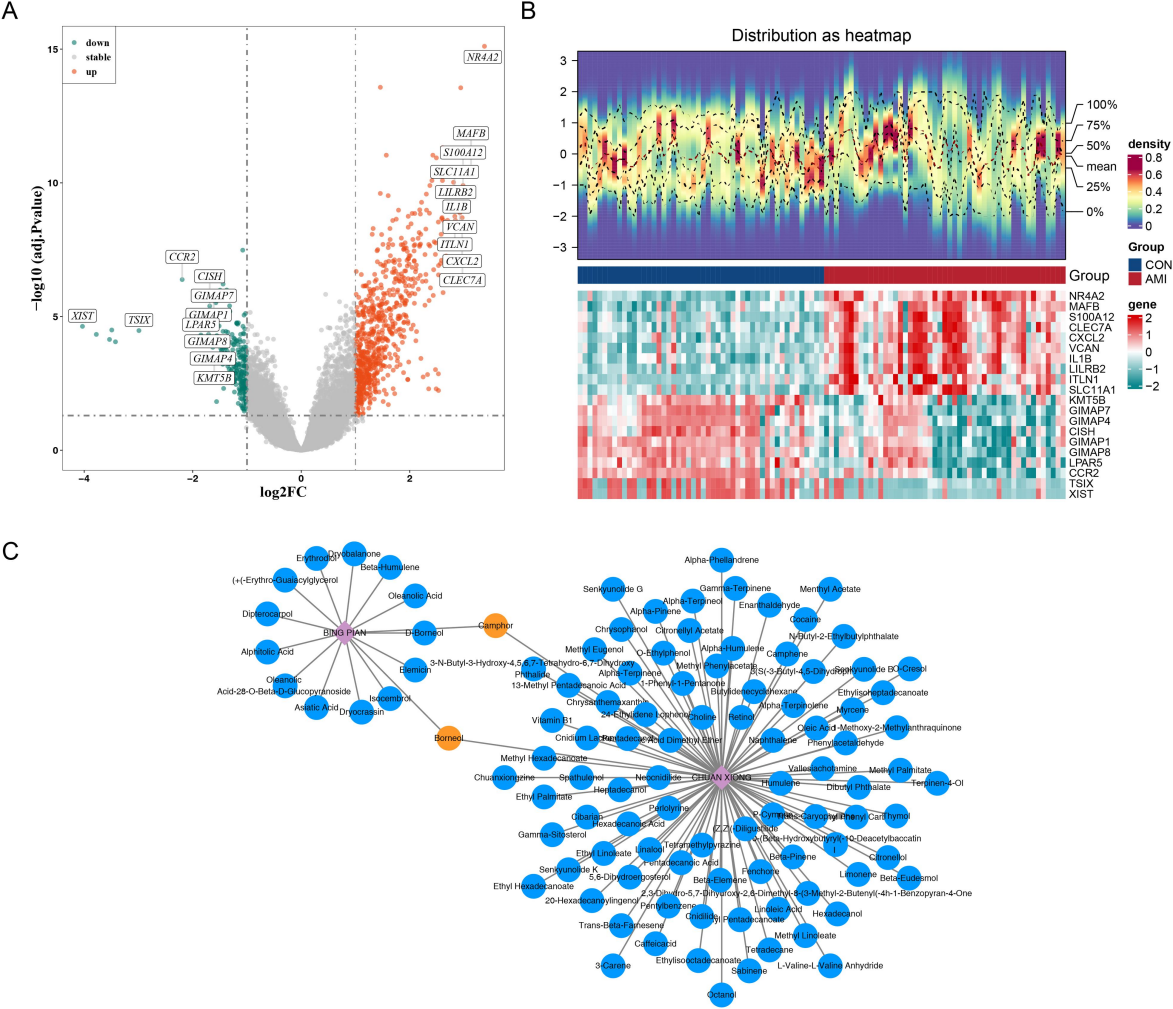
Identification of differentially expressed genes and construction of chuanXiong-bingPian-active components network. **(A)** Volcano plot of differentially expressed genes between AMI and control samples in training cohort, showing the distribution of up-regulated and down-regulated genes; **(B)** Heatmap of differentially expressed genes, showing the expression patterns of 689 DEGs in AMI and control samples; **(C)** ChuanXiong-BingPian-active components interaction network with 106 nodes and 106 edges, showing 91 compounds for ChuanXiong and 15 compounds for BingPian, with Borneol and Camphor as common active ingredients.

Primarily, we conducted a differentially expressed analysis comparing AMI and control samples within the training cohort. In all, 689 DEGs were screened out, with 488 genes being upregulated and 201 genes being downregulated (Figures 1A,B). Following this, we constructed a network to represent interactions among ChuanXiong, BingPian, and their active components, resulting in 106 nodes and 106 edges. The analysis predicted 91 compounds for ChuanXiong and 15 compounds for BingPian, revealing that Borneol and Camphor were active ingredients that interacted with both drugs (Figure 1C).

### 3.2 Totally 44 DE-TGs could influence the development of AMI through immune-related functions and pathways

**Figure 2:**
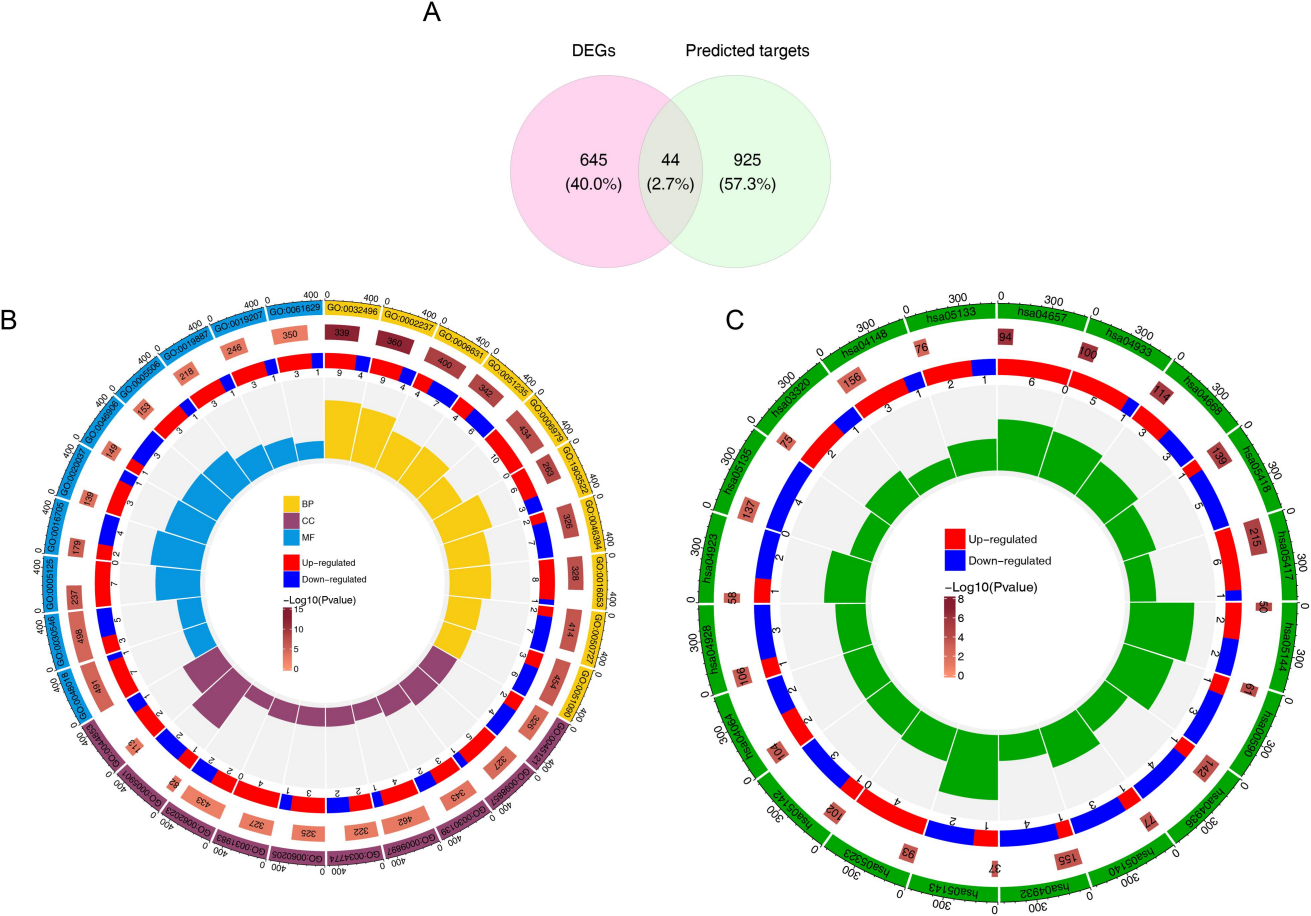
Identification and enrichment analysis of differentially expressed target genes. **(A)** Venn diagram showing the overlap of 689 DEGs and 969 predicted target genes, resulting in 44 differentially expressed target genes (DE-TGs); **(B)** Gene Ontology (GO) enrichment analysis of DE-TGs, including 1,253 GO entries; **(C)** Kyoto Encyclopedia of Genes and Genomes (KEGG) pathway enrichment analysis of DE-TGs, identifying 200 KEGG pathways.

Totally 44 DE-TGs were obtained by overlapping 689 DEGs and 969 predicted target genes (Figure 2A and Supplementary Data Sheet 2). Subsequently, 1,253 GO entries were identified, including 1,128 in BP category (like “response to lipopolysaccharide”), 29 in CC category (such as “membrane raft”), and 96 in MF category (e.g., “cytokine activity”) (Figure 2B and Supplementary Data Sheet 3). A sum of 200 KEGG pathways were discovered, comprising pathways such as the “IL-17 signaling pathway” and “TNF signaling pathway” (Figure 2C and Supplementary Data Sheet 4). This indicated that DE-TGs might impact the onset and development of AMI through immune and inflammation-related biological functions, as well as signaling pathways associated with immune regulation and cell signaling.

### 3.3 Discovery of 11 genes exhibiting causal correlations with AMI

**Figure 3:**
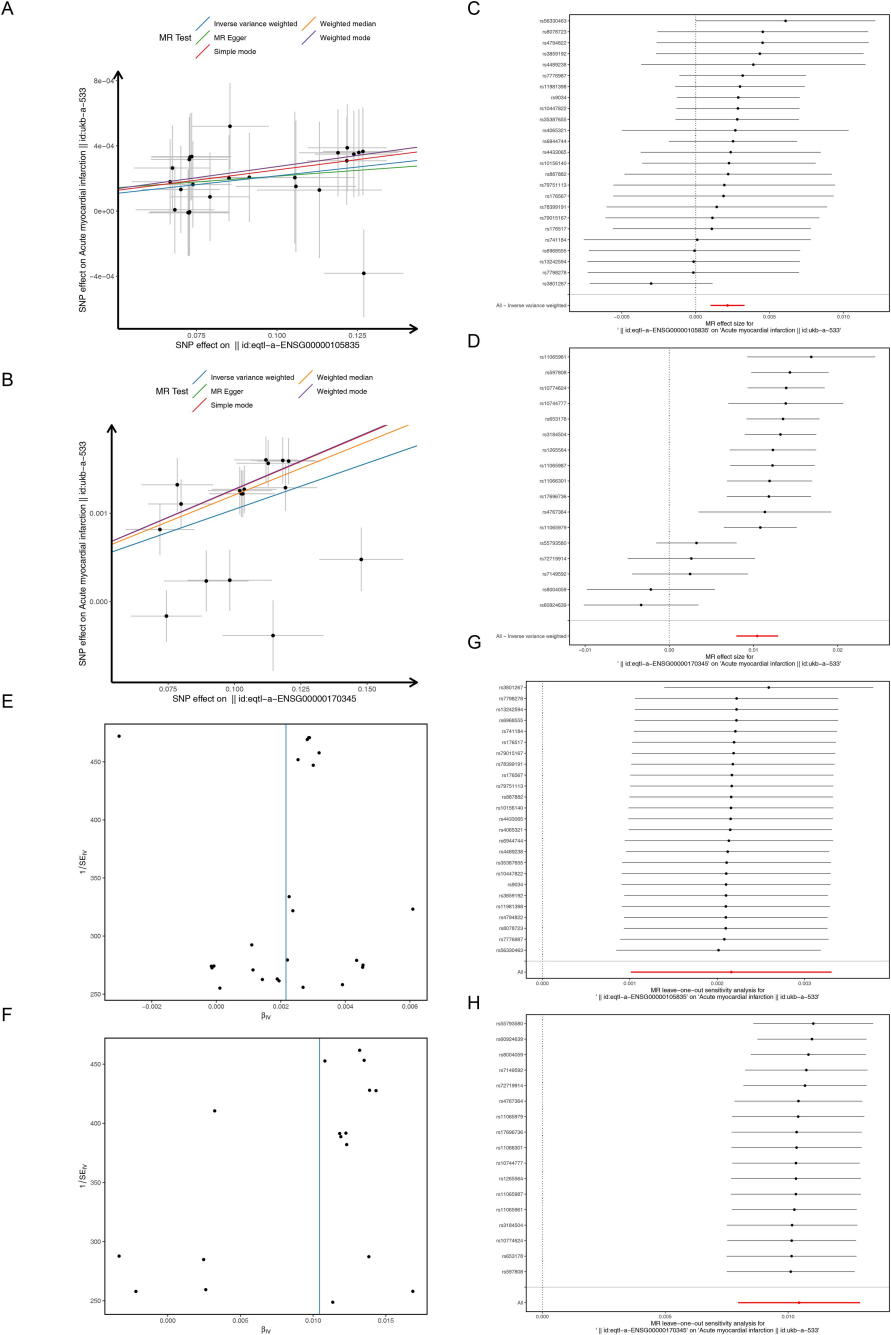
Mendelian randomization analysis identifies genes causally associated with AMI. **(A,B)** Scatter plots of NAMPT and FOS showing positive significant correlation with AMI, with positive slopes indicating they are risk factors for AMI; **(C,D)** Forest plots showing significant effects of IVW model for NAMPT and FOS (effect size > 0); **(E,F)** Funnel plots showing that MR analysis adheres to Mendel's second law of random grouping; **(G,H)** Leave-one-out sensitivity analysis showing that after gradually eliminating each SNP, the effect of remaining SNPs on outcome variables did not significantly change, indicating the reliability of MR analysis.

Overall, we identified 18 genes as candidate exposure factors, among which five genes were protective factors (OR < 1), whereas 13 genes were risk factors (OR > 1) (Table 1). The results for NAMPT and FOS were highlighted, while the others were presented in the Supplementary material. Of critical importance, NAMPT and FOS were risk factors for AMI. Scatter plots demonstrated a positive significant correlation of both NAMPT and FOS with AMI, their slopes were all positive, further indicating that they were a risk factor for AMI (Figures 3A,B, Supplementary Figure 1), a notable effect of the IVW model was evidenced (effect size of NAMPT, and FOS > 0) (Figures 3C,D, Supplementary Figure 2), and our MR analysis adhered to Mendel's law of independent assortment (Figures 3E,F, Supplementary Figure 3).

**Table 1:** Association of 18 candidate exposure factor genes identified by mendelian randomization analysis with AMI.

outcome	exposure	gene	nsnp	pval	or	or_lci95	or_uci95
Acute myocardial infarction	eqtl-a-ENSG00000011198	ABHD5	58	0.0000	1.0012	1.0008	1.0016
	eqtl-a-ENSG00000070495	JMJD6	30	0.0002	1.0006	1.0003	1.0010
	eqtl-a-ENSG00000070831	CDC42	63	0.0467	1.0003	1.0000	1.0006
	eqtl-a-ENSG00000095303	PTGS1	40	0.0322	0.9994	0.9988	0.9999
	eqtl-a-ENSG00000105835	NAMPT	25	0.0002	1.0022	1.0010	1.0033
	eqtl-a-ENSG00000111275	ALDH2	153	0.0000	1.0008	1.0005	1.0012
	eqtl-a-ENSG00000113448	PDE4D	31	0.0001	1.0016	1.0008	1.0023
	eqtl-a-ENSG00000118402	ELOVL4	63	0.0000	1.0012	1.0008	1.0016
	eqtl-a-ENSG00000124762	CDKN1A	42	0.0168	0.9990	0.9981	0.9998
	eqtl-a-ENSG00000145284	SCD5	55	0.0000	1.0004	1.0003	1.0006
	eqtl-a-ENSG00000147459	DOCK5	48	0.0179	0.9994	0.9989	0.9999
	eqtl-a-ENSG00000165029	ABCA1	52	0.0123	0.9990	0.9981	0.9998
	eqtl-a-ENSG00000165092	ALDH1A1	156	0.0000	1.0007	1.0005	1.0009
	eqtl-a-ENSG00000170345	FOS	17	0.0000	1.0105	1.0080	1.0130
	eqtl-a-ENSG00000176624	MEX3C	25	0.0007	0.9987	0.9980	0.9995
	eqtl-a-ENSG00000176845	METRNL	12	0.0026	1.0018	1.0006	1.0030
	eqtl-a-ENSG00000196715	VKORC1L1	169	0.0000	1.0005	1.0004	1.0006
	eqtl-a-ENSG00000232810	TNF	98	0.0000	1.0026	1.0019	1.0032

Next, the dependability of the MR analytical pipeline was assessed. Clearly and unequivocally, Cochran's *Q* test indicated no heterogeneity (*P* > 0.05) within samples (Table 2), while horizontal pleiotropy test suggested that ABHD5, ALDH2, ELOVL4, CDKN1A, DOCK5, MEX3C, and TNF had confounding factors, thus warranting their exclusion from further data analysis (*P* < 0.05) (Table 2). Following the gradual exclusion of each SNP one by one, the influence of the remaining SNPs on outcome variables remained largely unchanged, an indication of the MR analysis's stability (Figures 3G,H, Supplementary Figure 4). In conclusion, we identified 11 genes (JMJD6, CDC42, PTGS1, NAMPT, PDE4D, SCD5, ABCA1, ALDH1A1, FOS, METRNL, and VKORC1L1) with causal associations to AMI, which were subsequently used for further analysis.

**Table 2:** Sensitivity analysis results of mendelian randomization analysis.

outcome	exposure	gene	mr_pleiotropy_test Q_pval	mr_heterogeneity pval	Mrpresso pval
Acute myocardial infarction	eqtl-a-ENSG00000011198	ABHD5	0.8592	0.0016	0.835
	eqtl-a-ENSG00000070495	JMJD6	0.9364	0.0923	0.958
	eqtl-a-ENSG00000070831	CDC42	0.5028	0.0994	0.532
	eqtl-a-ENSG00000095303	PTGS1	0.2182	0.2823	0.155
	eqtl-a-ENSG00000105835	NAMPT	0.9859	0.753	0.978
	eqtl-a-ENSG00000111275	ALDH2	0	0	0.232
	eqtl-a-ENSG00000113448	PDE4D	0.9997	0.2772	1
	eqtl-a-ENSG00000118402	ELOVL4	0.4147	0.0002	0.117
	eqtl-a-ENSG00000124762	CDKN1A	0.0018	0.0131	0.001
	eqtl-a-ENSG00000145284	SCD5	0.4158	0.2183	0.135
	eqtl-a-ENSG00000147459	DOCK5	0.0171	0.7649	0.009
	eqtl-a-ENSG00000165029	ABCA1	0.051	0.0587	0.715
	eqtl-a-ENSG00000165092	ALDH1A1	0.9572	0.9199	0.967
	eqtl-a-ENSG00000170345	FOS	0	0.9976	0.242
	eqtl-a-ENSG00000176624	MEX3C	0.9828	0.0403	0.991
	eqtl-a-ENSG00000176845	METRNL	0.7643	0.1748	0.776
	eqtl-a-ENSG00000196715	VKORC1L1	1	0.1887	1
	eqtl-a-ENSG00000232810	TNF	0	0.0012	0.032

### 3.4 Key target genes were confirmed to be NAMPT and FOS

**Figure 4:**
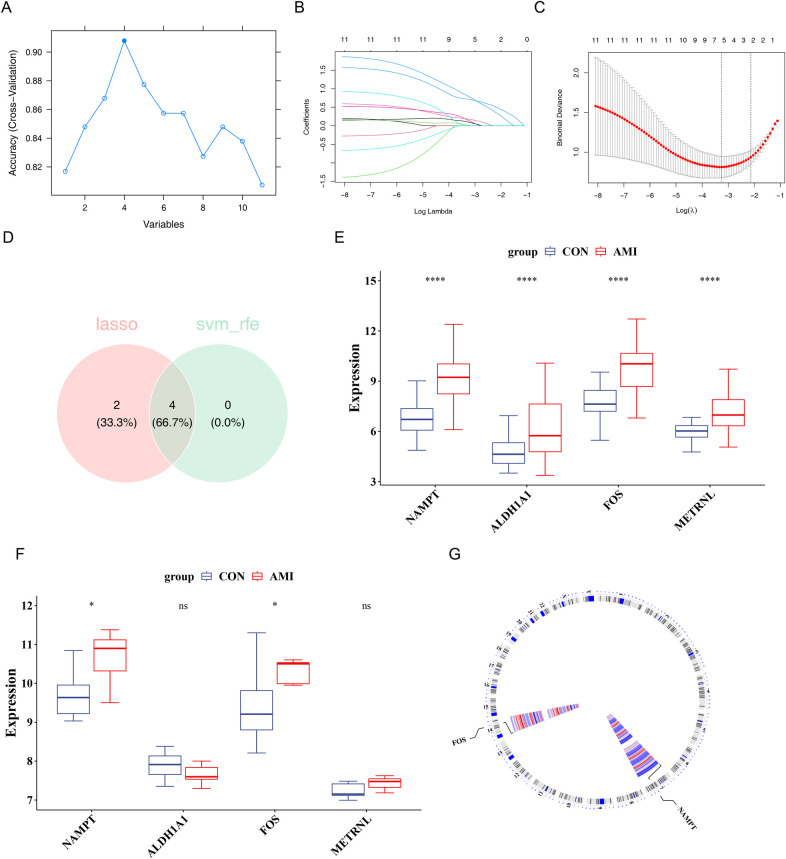
Identification of key target genes by machine learning algorithms. **(A)** Support vector machine-recursive feature elimination (SVM-RFE) algorithm screened out NAMPT, ALDH1A1, FOS, and METRNL; **(B,C)** Least absolute shrinkage and selection operator (LASSO) algorithm identified NAMPT, SCD5, ABCA1, ALDH1A1, FOS, and METRNL; **(D)** Overlapping results of two machine learning algorithms revealed four candidate key target genes: NAMPT, ALDH1A1, FOS, and METRNL; **(E)** Expression levels of four candidate key genes showing significantly higher expression in AMI samples in training cohort. ****p < 0.0001; **(F)** No significant difference between AMI and control samples in validation cohort for ALDH1A1 and METRNL, while NAMPT and FOS showed significantly elevated expression.*p < 0.05, and ns indicates no significance; **(G)** Chromosomal distribution of NAMPT and FOS, located on chromosome 7 and 14, respectively.

We employed SVM-RFE and the LASSO algorithm to further identify key target genes from the previously obtained 11 genes. Explicitly, in the SVM-RFE algorithm, NAMPT, ALDH1A1, FOS, and METRNL were obtained (Figure 4A), as well as NAMPT, SCD5, ABCA1, ALDH1A1, FOS, and METRNL were gained via the LASSO algorithm (Figures 4B,C). Besides, the overlap of results yielded four candidate key target genes: NAMPT, ALDH1A1, FOS, and METRNL (Figure 4D). Within the training cohort, AMI samples showed notably increased expression of these candidate key genes (*P* < 0.0001) (Figure 4E), nevertheless, no notable difference was identified in ALDH1A1 and METRNL expression between 2 groups in the validation cohort (*P* < 0.05) (Figure 4F). Consequently, we selected NAMPT and FOS as key target genes for subsequently analysis. Besides, NAMPT and FOS were distributed on chromosomes 7 and 14, respectively (Figure 4G).

### 3.5 NAMPT and FOS demonstrated enrichment in pathways related to immune response, cell migration, and communication

**Figure 5:**
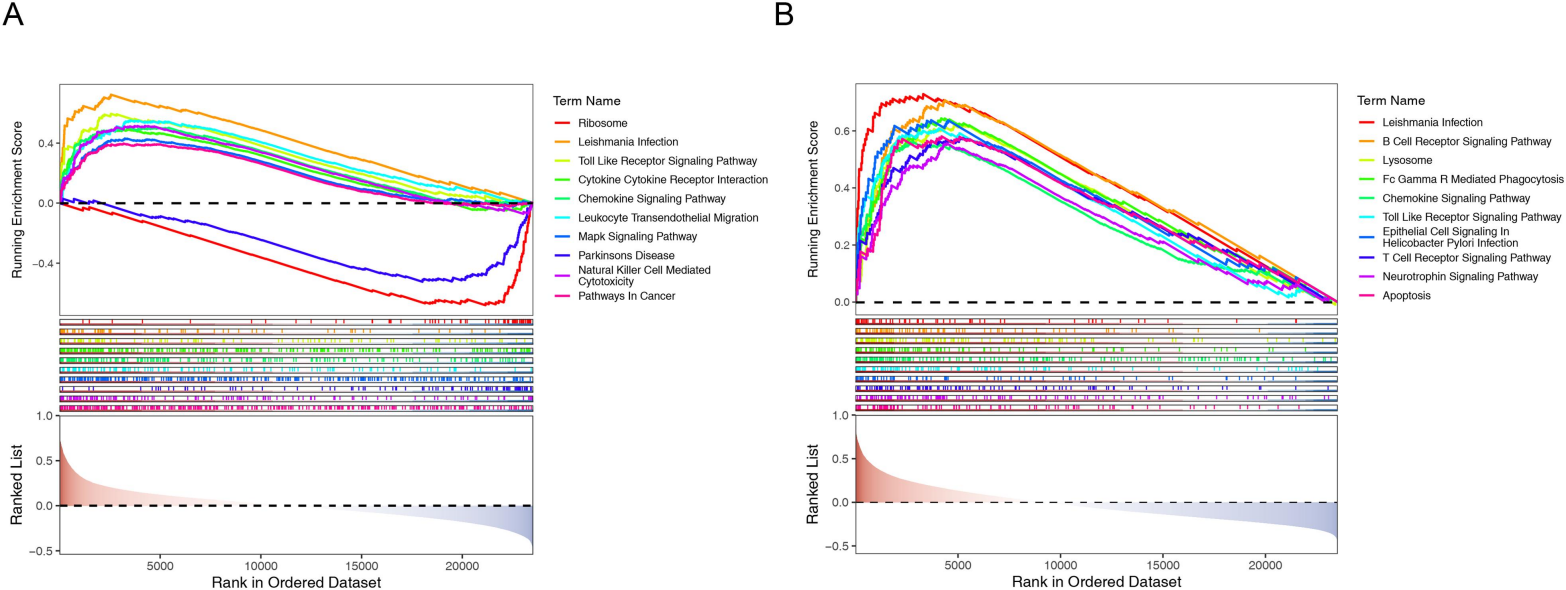
Gene Set enrichment analysis (GSEA) of key target genes. **(A,B)** GSEA analysis of NAMPT and FOS.

To this end, we conducted GSEA for key target genes. Noticeably, we found that “Toll-Like Receptor Signaling Pathway”, “Leishmania Infection”, and “Chemokine Signaling Pathway” were common pathway for NAMPT and FOS (Figures 5A,B). Intriguingly, these genes identified were enriched within the relevant pathways linked to cell migration and cellular communication processes, which coincided with our results described above. It might imply that the key target genes corresponding to active components of SJP could influence AMI development through these pathways.

### 3.6 Immune response of AMI patients

**Figure 6:**
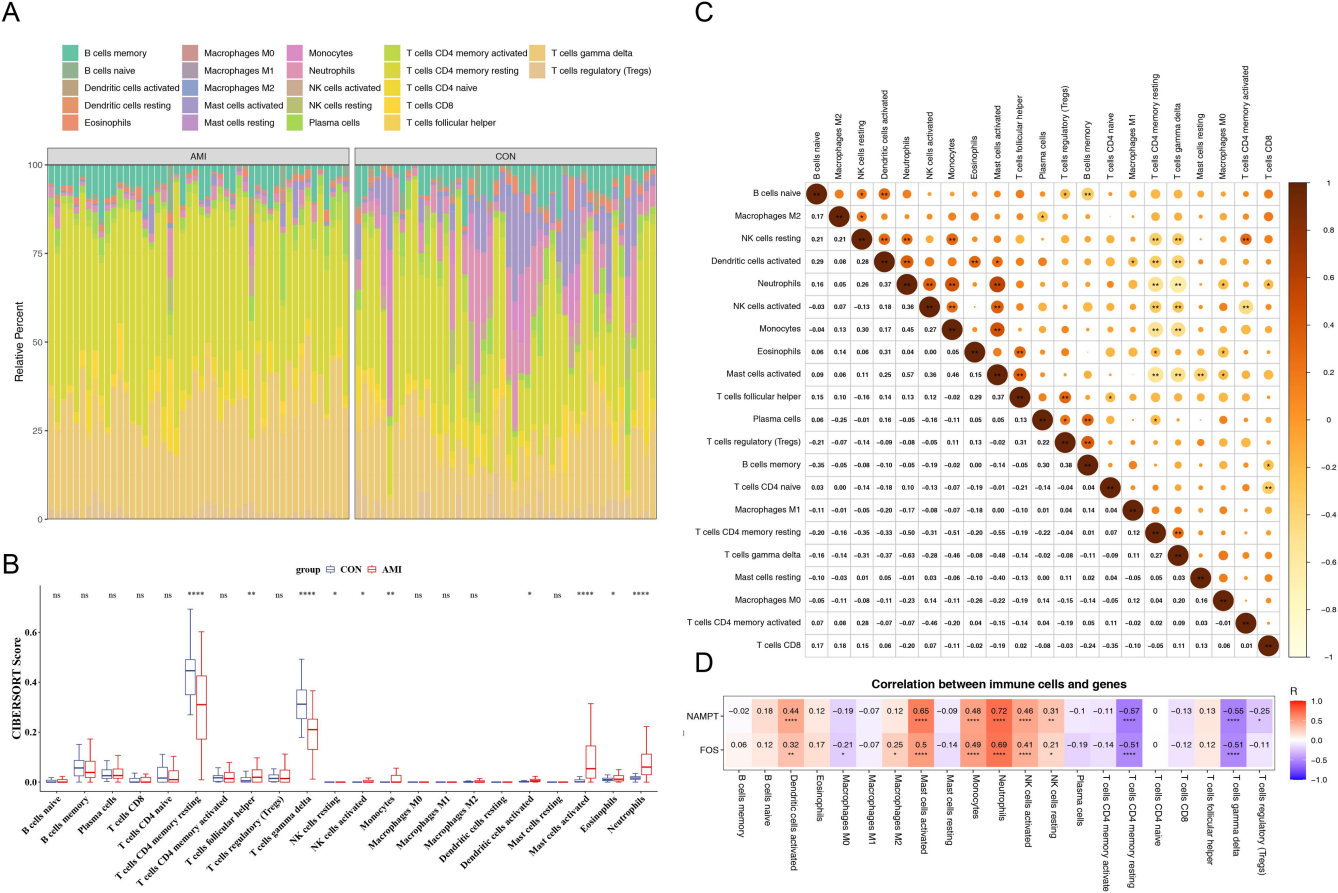
Immune infiltration analysis. **(A)** Stacked bar chart displaying the proportion of 22 immune cells in 48 AMI and 48 control samples; **(B)** Comparative analysis of differential immune cells showing significantly higher proportions of activated Dendritic cells, Eosinophils, activated Mast cells, Monocytes, activated NK cells, resting NK cells, Neutrophils, and T follicular helper cells in AMI samples (*P* < 0.05), while resting memory CD4T cells and gamma delta T cells showed decreased proportions (*P* < 0.0001); **(C)** Correlation analysis among different immune cells showing strongest positive correlation between activated Mast cells and Neutrophils (*P* < 0.01, co*r* = 0.57), and strongest negative correlation between gamma delta T cells and Neutrophils (*P* < 0.01, cor = −0.63); **(D)** Correlation analysis between differential immune cells and key target genes showing strongest positive correlation between NAMPT and Neutrophils (*P* < 0.0001, co*r* = 0.65), and strongest negative correlation between NAMPT and resting memory CD4T cells (*P* < 0.0001, cor = −0.57). *p < 0.05, **p < 0.01, ***p < 0.001, ****p < 0.0001, and ns indicates no significance.

Enrichment analysis results indicated an association between NAMPT and FOS with immune-related pathways, thereby motivating an investigation into immune infiltration dynamics in the AMI immune microenvironment. The 48 AMI and 48 control samples were included in the immune infiltration analysis. The stacked bar chart displayed the proportion of 22 cells (Figure 6A). Among these, the proportion of Eosinophils, Monocytes, Neutrophils, etc. were significantly higher in disease samples (*P* < 0.05) (Figure 6B).

Moreover, we observed varying degrees of correlation within different immune cells (Figure 6C). Activated mast cells showed the strongest positive correlation with neutrophils (*P* < 0.01 and co*r* = 0.57). These genes showed strong associations with a wide range of differential immune cells. The supreme positive correlation was observed from NAMPT and Neutrophils (*P* < 0.0001 and co*r* = 0.65) (Figure 6D). The results suggested that key target genes might offer a holistic characterization, which was critical for AMI.

### 3.7 Network and molecular docking

**Figure 7:**
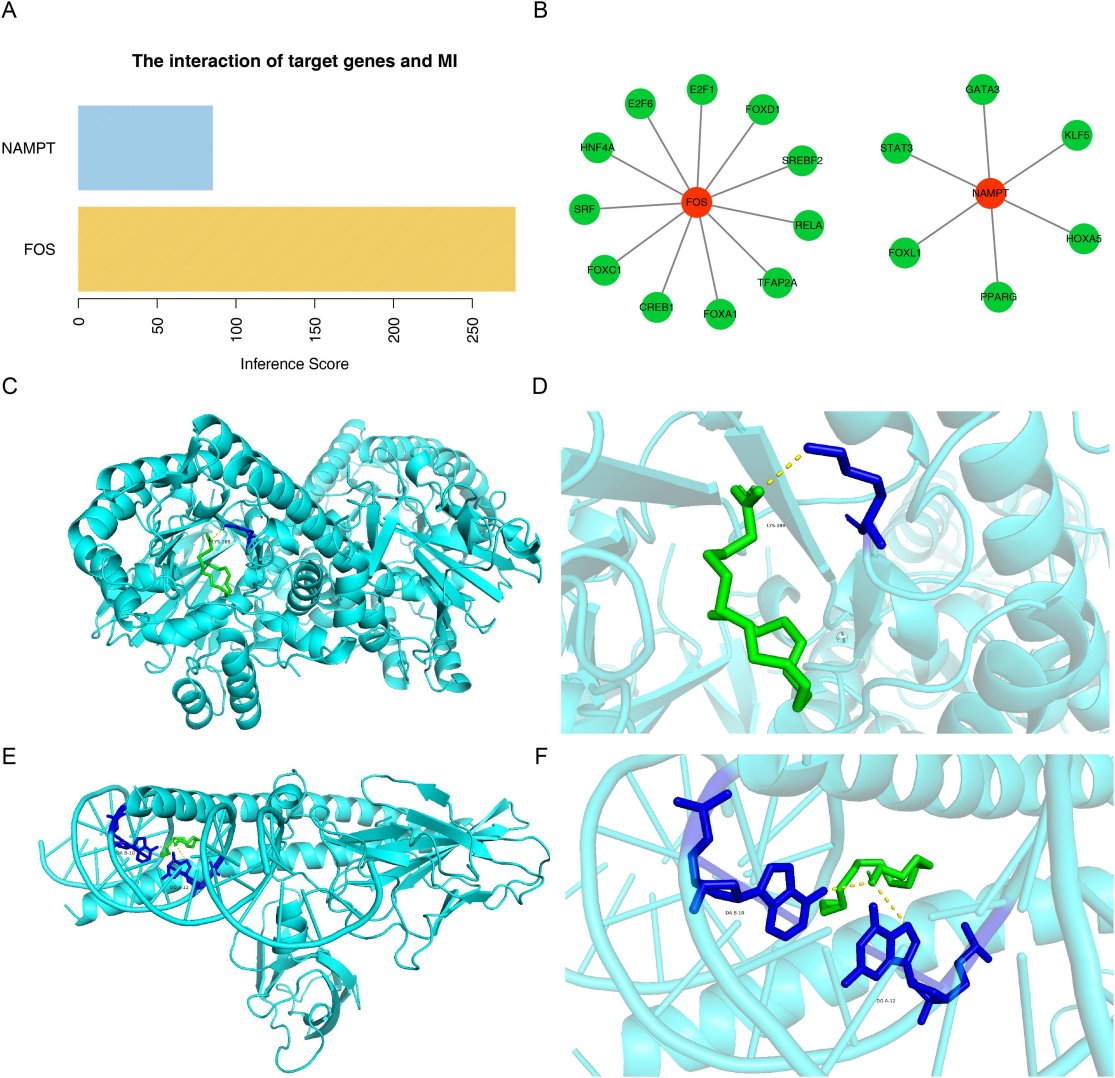
Disease association, transcription factor regulatory network and molecular docking analysis. **(A)** Inference scores between NAMPT and FOS and AMI were 85.23 and 277.31, respectively, indicating a strong correlation between these key target genes and AMI, particularly FOS; **(B)** Transcription factor (TF)-mRNA regulatory network showing 14 TFs regulating key target genes, with NAMPT regulated by six TFs and FOS by 11; **(C,D)** Molecular docking analysis of NAMPT with Oleic Acid showing binding energy of −5.9 kcal/mol, with LYS-189 residue exhibiting hydrogen bonding interactions with Oleic Acid; **(E,F)** Molecular docking analysis of FOS with Pentadecanol showing binding energy of −5.4 kcal/mol, with DA B-10 and DG A-12 residues showing hydrogen bonding interactions with Pentadecanol.

The inference scores between NAMPT and FOS and AMI were 85.23 and 277.31, respectively, indicating a strong correlation between these key target genes and AMI, particularly FOS (Figure 7A). Further, a total of 14 TFs regulating key target genes in the JASPAR database were predicted, with NAMPT being regulated by six TFs and FOS by 11 (Figure 7B).

Molecular docking showed that NAMPT could bind to Oleic Acid (−5.9  kcal/mol), and the LYS-189 residue exhibited hydrogen bonding interactions with the Oleic Acid (Figures 7C,D). Meanwhile, FOS could bind to Pentadecanol (−5.4  kcal/mol), and the DA B-10, DG A-12 residue showed hydrogen bonding interactions with the Pentadecanol (Figures 7E,F).

### 3.8 SJP reduced the expression of FOS in MI, but not NAMPT

**Figure 8:**
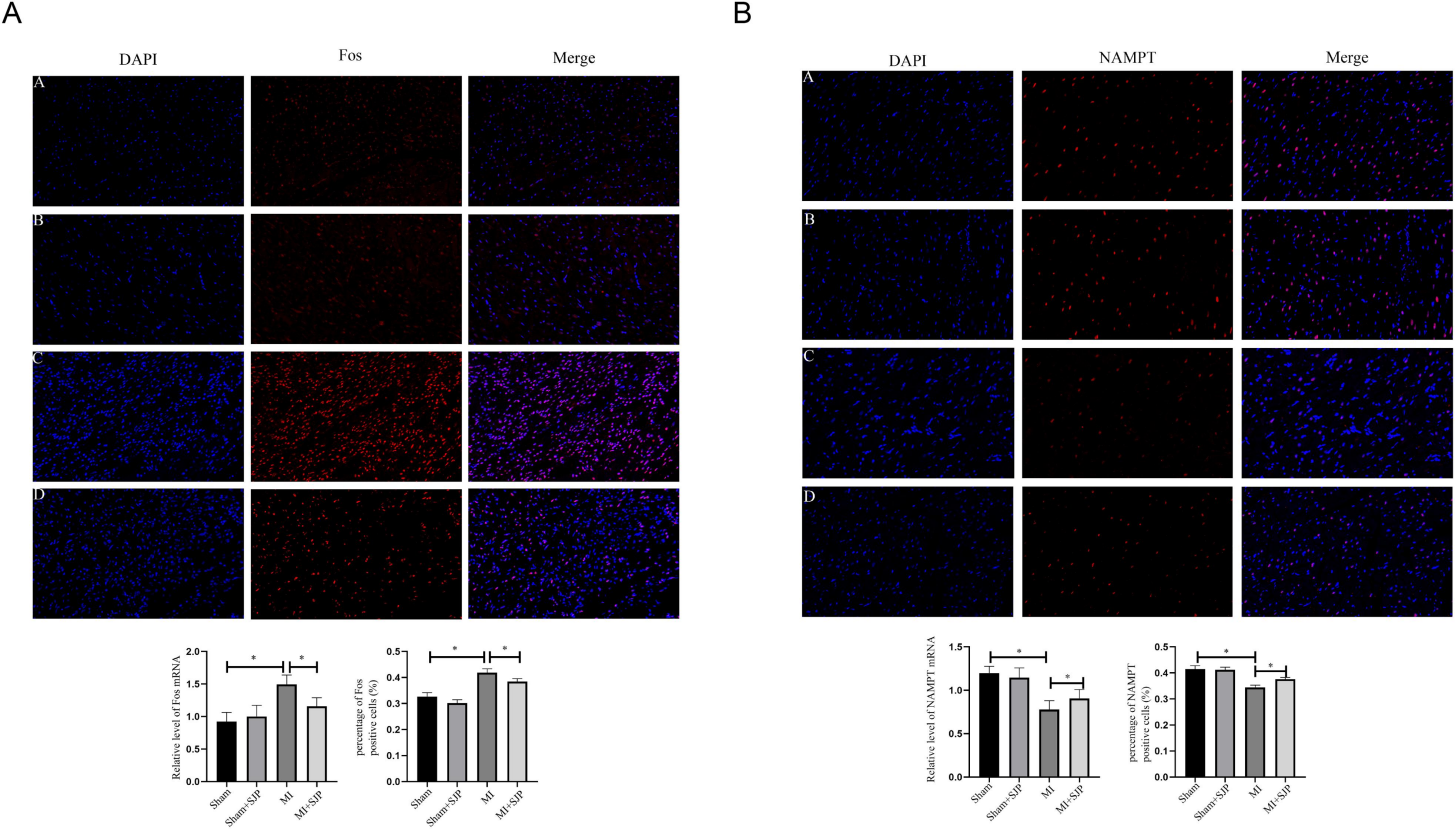
Effects of suxiao jiuxin pill on FOS and NAMPT expression. **(A)** Representative immunofluorescence staining images of FOS protein in myocardial tissues from four groups of rats. DAPI staining shows nuclei (blue), FOS antibody staining shows FOS protein expression (red), and Merge shows merged images. Compared with Sham group, FOS protein expression was significantly increased in myocardial tissue of MI group; compared with MI group, FOS expression was downregulated in MI + SJP group; **(B)** Representative immunofluorescence staining images of NAMPT protein in myocardial tissues from four groups of rats. DAPI staining shows nuclei (blue), NAMPT antibody staining shows NAMPT protein expression (red), and Merge shows merged images. Compared with the Sham group, NAMPT protein expression in myocardial tissue was significantly decreased in the MI group; compared with the MI group, NAMPT expression was upregulated in the MI + SJP group. *p < 0.05.

The MI group exhibited significantly higher Fos expression levels compared with the sham group, as evidenced by PCR and immunofluorescence analyses (Figure 8A). Administration of SJP, however, led to a reduction in Fos levels. Consistent with our earlier discoveries, this suggests that SJP exerts a protective effect against MI by inhibiting Fos expression. Notably, NAMPT expression displayed the inverse pattern (Figure 8B).

### 3.9 SJP improves cardiac function in established AMI models

**Figure 9:**
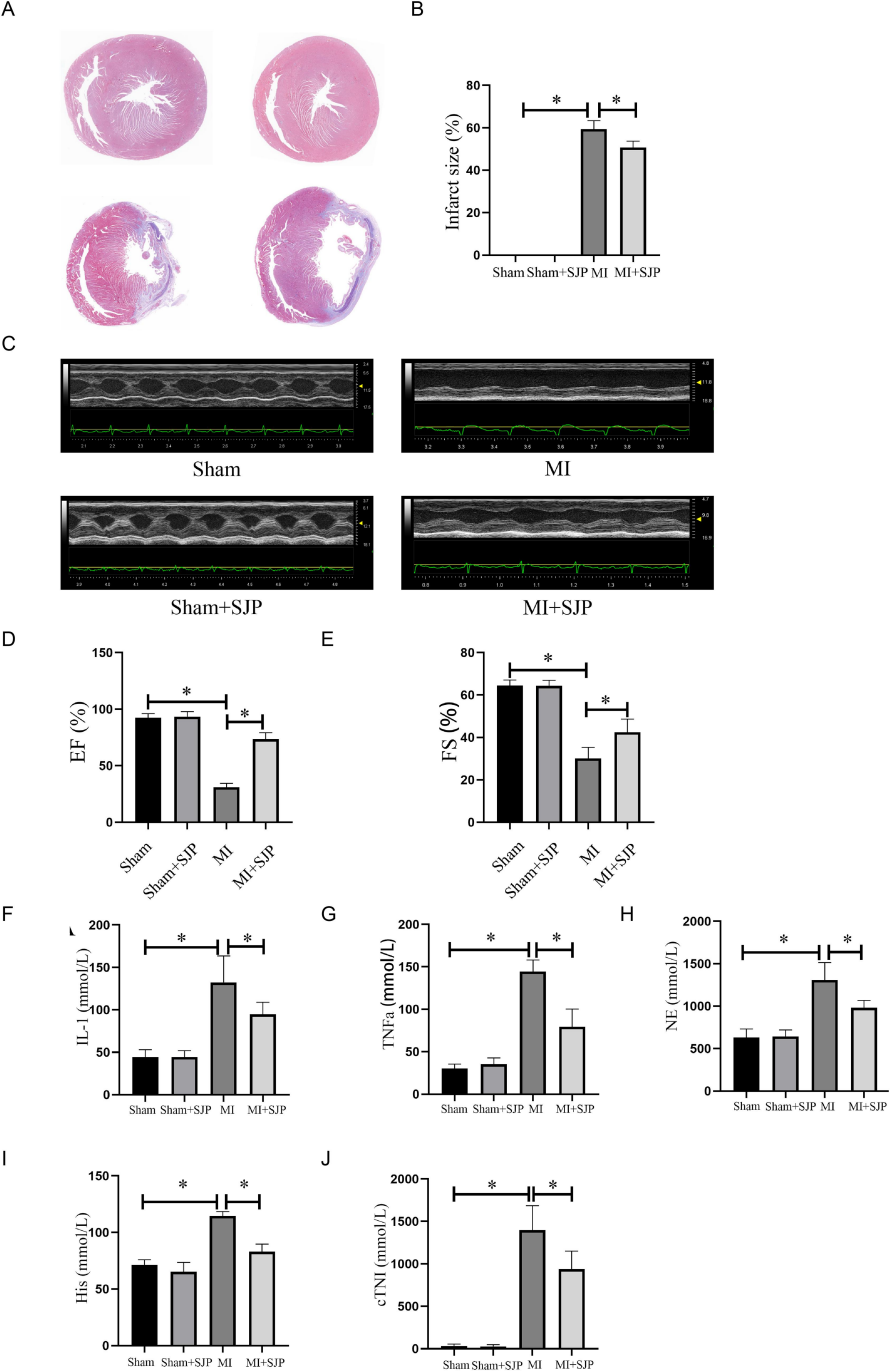
Suxiao jiuxin pill improves infarct area and cardiac function in MI. **(A,B)** Masson staining showing infarct area of cardiac tissue, with MI + SJP group showing significant reduction in infarct area compared to MI group alone; **(C–E)** Hemodynamic analysis showing decreased left ventricular EF and FS in MI group rats after surgery compared to Sham group; Suxiao Jiuxin Pill improved these indicators compared to MI group alone (*P* < 0.01); **(F–J)** ELISA assay showing that IL-1, NE, His and cTNI were significantly reduced in MI + SJP group compared to MI group. *p < 0.05.

Coronary artery ligation induced pale, atrophic ventricular walls, confirming the successful construction of a rat AMI model. Masson staining was performed to quantify myocardial infarct area, which revealed a marked reduction in the MI + SJP group relative to the MI-only cohort (Figures 9A,B). Cardiac function assessments demonstrated that left ventricular EF and FS were significantly decreased in MI rats compared with sham-operated controls. Notably, SJP administration reversed these functional impairments in MI models (*P* < 0.01; Figures 9C–E). Collectively, these data verify that Suxao Jiuxin Pill attenuates myocardial infarct size and ameliorates cardiac function post-MI.

### 3.10 Inflammatory markers were decreased in the AMI with SJP pretreatment group

ELISA assays were conducted to characterize the anti-inflammatory properties of SJP. We found that concentrations of IL-1, TNF*α*, NE, His, and cTNI were substantially reduced in the MI + SJP group relative to the MI group (Figures 9F–J).

## 4 Discussion

Globally, AMI persists as a major worldwide health threat, and its inflammatory response plays a critical pathological role in post-infarction cardiac remodeling and dysfunction (54, 55). Despite significant progress in reperfusion therapy, the persistent inflammatory cascade after AMI still poses a major treatment challenge. To address this unmet need for adjunctive treatments (56), a study was undertaken to elucidate the comprehensive molecular mechanisms underlying the therapeutic effect of Suxiao Jiuxin Pill (SJP) on AMI, integrating network pharmacology, Mendelian randomization analysis, and experimental verification. The research results confirmed NAMPT and FOS as the core therapeutic targets, with FOS showing sustained upregulation in AMI samples, significantly downregulated after SJP intervention, and accompanied by improved heart function and reduced inflammatory response observed in a rat AMI model.

Representing a promising target for AMI intervention, NAMPT (visfatin) catalyzes the rate-limiting step in NAD + production—a metabolic pathway closely implicated in the development of multiple cardiovascular diseases (57, 58). Our Mendelian randomized analysis shows that there is a causal relationship between the increased expression of NAMPT and the increased risk of AMI, which is consistent with the recent research results on NAMPT gene variation and the susceptibility to myocardial infarction (59). This study observed a strong positive correlation between NAMPT and neutrophil infiltration, which confirmed the function of NAMPT in promoting neutrophil recruitment and activation in inflammatory state (60). Neutrophils, as the earliest immune cells to infiltrate the infarcted myocardium, release proteolytic enzymes and reactive oxygen species, which mediate tissue damage and start the repair process at the same time (61). However, the negative correlation between NAMPT and resting memory CD4 + T cells suggests that there is a complex interaction between innate immunity and adaptive immunity in AMI, and it is necessary to further explore the specific mechanism of NAMPT regulating different immune cell populations in the process of myocardial injury.

FOS, as a fundamental element of the activator protein 1 (AP-1) transcriptional complex. is most consistent verification target in this study. The experiment confirmed that FOS expression in AMI samples was up-regulated and down-regulated after SJP intervention, which provided strong evidence for its therapeutic target position. This protein critically influences the progression of cardiac remodeling through its modulation of key genes involved in inflammatory pathways, fibrotic development, and hypertrophic growth (62). Latest research shows that FOS participates in the inflammatory response of cardiovascular diseases by controlling the transcription of pro-inflammatory cytokines and chemokines (62). The discovery that SJP therapy can reduce FOS expression is of great value because the over-activation of FOS is closely related to poor cardiac remodeling and the progression of heart failure (63). The improvement of cardiac function and the decrease of inflammatory markers observed in animals treated with SJP further prove the clinical significance of targeted FOS in the treatment of AMI.

After identifying NAMPT and FOS as key target genes, gene set enrichment analysis revealed that they are predominantly enriched in pathways related to immune response, cell migration, and cellular communication, including the Toll-like receptor signaling pathway and chemokine signaling pathway. These pathways play critical roles in the pathophysiology of myocardial infarction, where a robust inflammatory response is triggered and proves essential for subsequent cardiac repair. Following myocardial injury, Toll-like receptor signaling is rapidly activated in the infarcted myocardium, initiating inflammatory cascades (54). Research has confirmed that Toll-like receptor 9 (TLR9) induces inflammatory responses during myocardial infarction by detecting immunogenic fragments of endogenous DNA (64). Concurrently, chemokine signaling orchestrates the recruitment and activation of immune cells to the injured myocardium, playing a critical function in inflammatory processes underlying diverse cardiovascular conditions, including myocardial infarction (65). This may mean that the key target genes corresponding to the active ingredients of SJP can affect the development of AMI by improving the inflammatory response.

To further elucidate the mechanisms underlying the therapeutic efficacy of Suxiao Jiuxin Pill (SJP) in AMI treatment, we performed molecular docking analysis to investigate the binding interactions between active ingredients and key target proteins. Molecular docking analysis revealed that NAMPT binds to oleic acid with a binding energy of −5.9 kcal/mol through hydrogen bonding with the LYS-189 residue, while FOS interacts with pentadecanol (binding energy: −5.4 kcal/mol) via hydrogen bonds involving DA B-10 and DG A-12 residues. These favorable binding interactions suggest potential mechanisms underlying SJP's therapeutic effects. Oleic acid, an unsaturated omega-9 fatty acid abundant in dietary sources and human tissues, is also one of the fatty acids with high content in human tissues and blood, which has an important impact on human health (66). Previous studies have demonstrated that oleic acid alleviates myocardial injury through multiple mechanisms, including antioxidant activity, regulation of cardiac metabolism, and attenuation of inflammatory responses (67–69).

To validate the results obtained through network pharmacology and molecular docking analysis, we used rat myocardial infarction model to carry out systematic experiments *in vivo*. PCR and immunofluorescence detection confirmed that FOS expression in acute myocardial infarction samples was significantly up-regulated, and it was significantly down-regulated after the intervention of SJPs, which was mutually confirmed with the prediction results of bioinformatics. This finding is consistent with the law revealed by previous studies-immediate and early genes such as FOS in infarcted myocardium will be rapidly induced and expressed, thus regulating inflammation and remodeling reaction (62). It is noteworthy that, as a key component of AP-1 transcription factor complex, FOS can regulate the expression of genes related to inflammation, fibrosis and myocardial hypertrophy (70). The intervention of SJP in reducing FOS expression suggests that this Chinese medicine may play a role by regulating excessive inflammatory signal pathway.

Echocardiographic evaluation showed that the cardiac function parameters of rats in SJP treatment group were significantly improved, comprising enhanced left ventricular ejection fraction and attenuated ventricular remodeling. This functional improvement is consistent with anti-inflammatory and cardioprotective effects mediated by FOS down-regulation (71, 72). Furthermore, through ELISA analysis, SJP treatment notably lowered systemic concentrations of the pro-inflammatory cytokines TNF-α, IL-1β, and IL-6. These molecules are markedly elevated during AMI's acute phase and serve as pivotal mediators of myocardial damage, impaired contractility, and adverse remodeling (73, 74). The simultaneous decline of inflammatory markers and the improvement of cardiac function strongly support the hypothesis that SJPs play a therapeutic role by regulating FOS-related inflammatory pathways. The above experimental verification builds a bridge between computational prediction and biological reality, and provides solid evidence for the multi-target anti-inflammatory mechanism of SJP in the treatment of acute myocardial infarction.

By integrating network pharmacology, Mendel randomization and experimental verification, this study reveals that SJPs can improve acute myocardial infarction mainly by targeting FOS to regulate inflammatory reaction, and expounds its new therapeutic mechanism. However, some limitations still need attention: the clinical applicability of bioinformatics findings demands validation in an expanded clinical cohort; Although FOS-mediated inflammatory regulation has been confirmed, the unconventional expression pattern of NAMPT suggests that there are other unexplored mechanisms. Subsequent studies ought to prioritize explaining timing regulation of NAMPT in different stages of myocardial infarction, conducting prospective clinical trials to verify the universality of the targeted FOS treatment effect of SJPs in different patient groups, and exploring the synergistic interaction of multi-active components to optimize the dosage regimen. These research directions are not only helpful to clarify overall mechanism of traditional Chinese medicine intervention, but also likely to provide theoretical guidance for the precision treatment of patients with acute myocardial infarction.

## 5 Conclusion

In conclusion, this study demonstrates that Suxiao Jiuxin Pill alleviates acute myocardial infarction primarily by downregulating the FOS gene. This action reduces inflammatory response, improves cardiac function, and decreases infarct size, providing mechanistic evidence for its clinical use as a complementary therapy.

## Data Availability

The original contributions presented in the study are included in the article/Supplementary Material, further inquiries can be directed to the corresponding author.

## Supplementary material

Supplementary Data Sheet 1 Active components of Suxiao Jiuxin Pill and their corresponding predicted target genes.

Supplementary Data Sheet 2 List of 44 differentially expressed target genes (DE-TGs).

Supplementary Data Sheet 3 Gene Ontology (GO) enrichment analysis results of differentially expressed target genes.

Supplementary Data Sheet 4 Kyoto Encyclopedia of Genes and Genomes (KEGG) pathway enrichment analysis results of differentially expressed target genes.

Supplementary Figure 1 Scatter plot of the Mendelian Randomization analysis. A positive slope indicates that the gene is a risk factor for AMI, while a negative slope indicates a protective factor.

Supplementary Figure 2 Forest plot of the Mendelian Randomization analysis. An effect size greater than 0 indicates a risk factor, whereas an effect size less than 0 indicates a protective factor.

Supplementary Figure 3 Funnel plot of the Mendelian Randomization analysis. A symmetrical distribution suggests the absence of significant horizontal pleiotropy in the analysis, which is consistent with Mendel's law of independent assortment.

Supplementary Figure 4 Leave-one-out sensitivity analysis plot. This plot displays the overall effect estimate on AMI from the remaining SNPs after sequentially excluding each individual SNP. The results remained stable, indicating that the analysis was notdisproportionately driven by any single SNP.
