# Supplementary figures and images for: Molecular mechanisms of suxiao jiuxin pills in ameliorating post-acute myocardial infarction inflammatory response: a combined network pharmacology, Mendelian randomization, and experimental validation study
Source: Front Cardiovasc Med. 2026 Jul 16;13:1852925. doi: 10.3389/fcvm.2026.1852925 (PMC13422419; doi:10.3389/fcvm.2026.1852925)

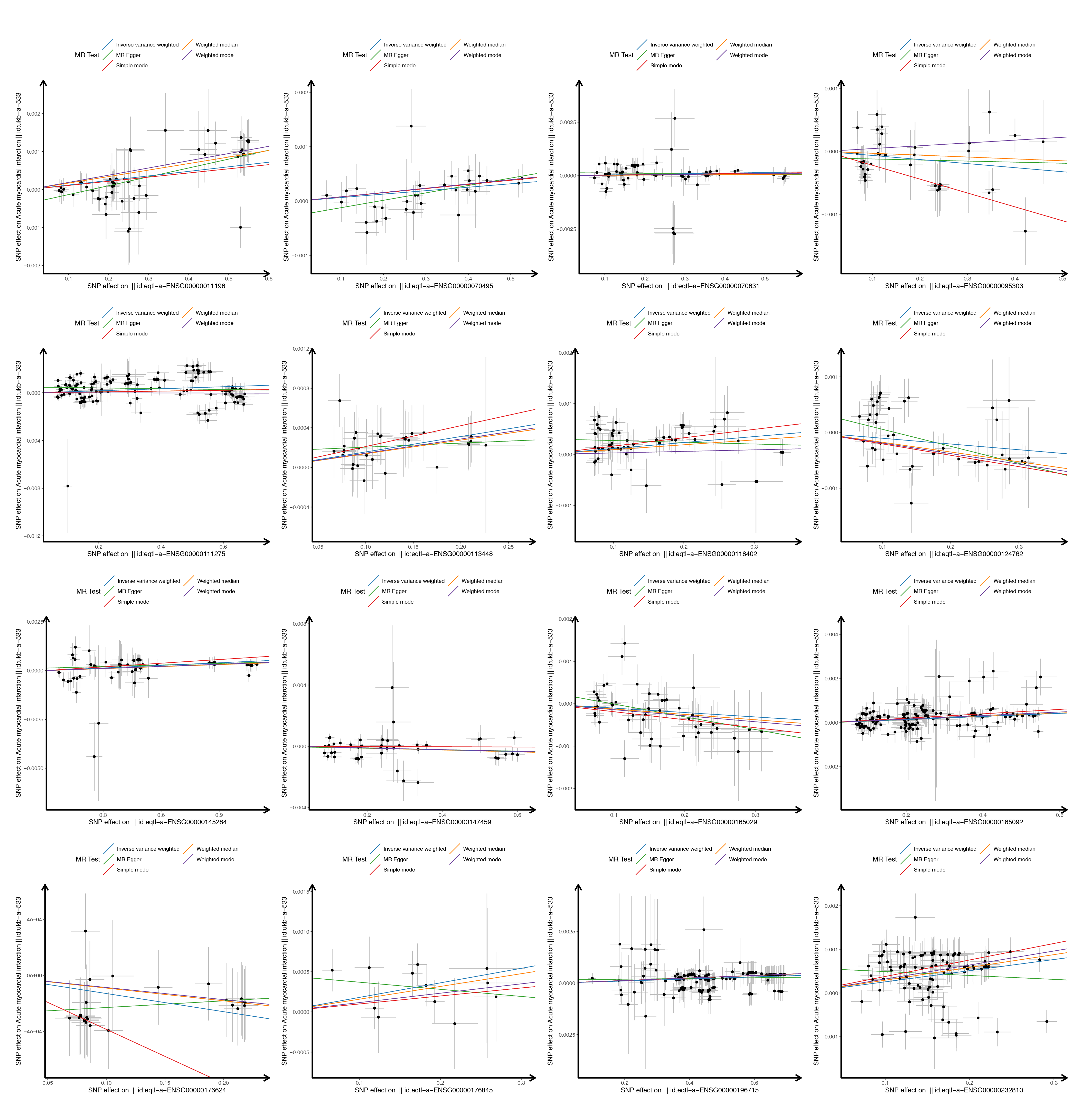

Supplement: Supplementary Figure 1 — Scatter plot of the Mendelian Randomization analysis. A positive slope indicates that the gene is a risk factor for AMI, while a negative slope indicates a protective factor. [file Image1.tif]

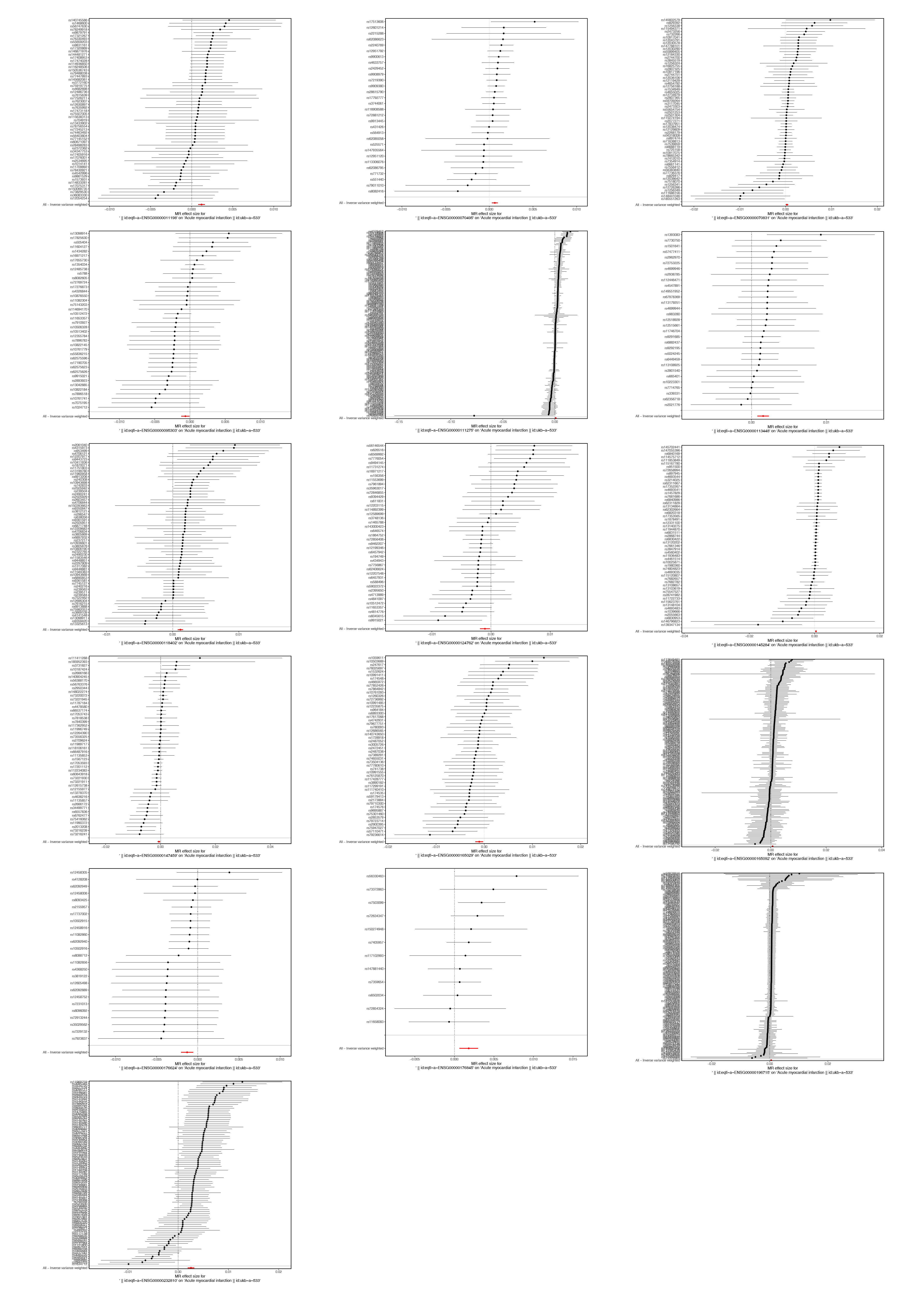

Supplement: Supplementary Figure 2 — Forest plot of the Mendelian Randomization analysis. An effect size greater than 0 indicates a risk factor, whereas an effect size less than 0 indicates a protective factor. [file Image2.tif]

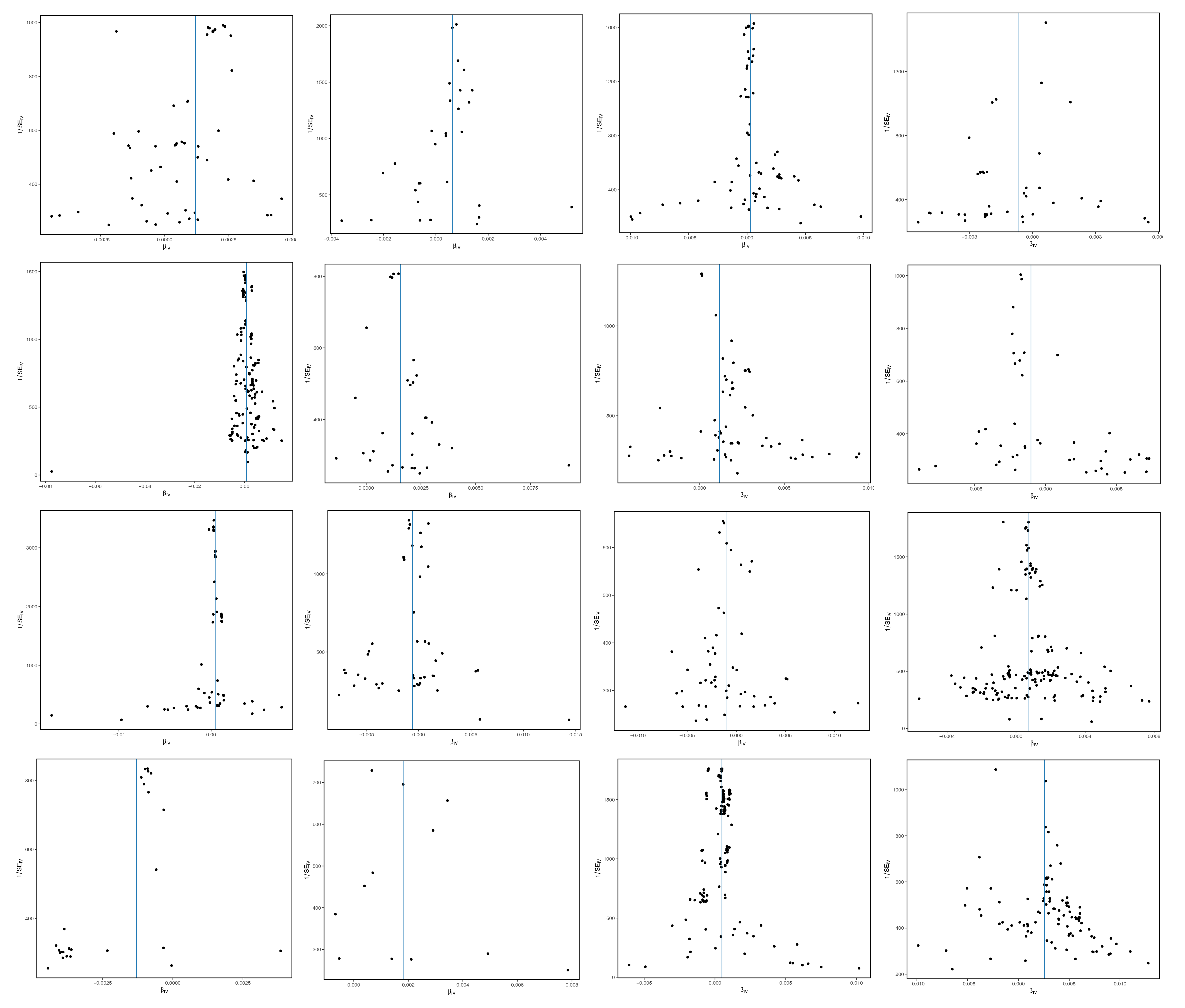

Supplement: Supplementary Figure 3 — Funnel plot of the Mendelian Randomization analysis. A symmetrical distribution suggests the absence of significant horizontal pleiotropy in the analysis, which is consistent with Mendel's law of independent assortment. [file Image3.tif]

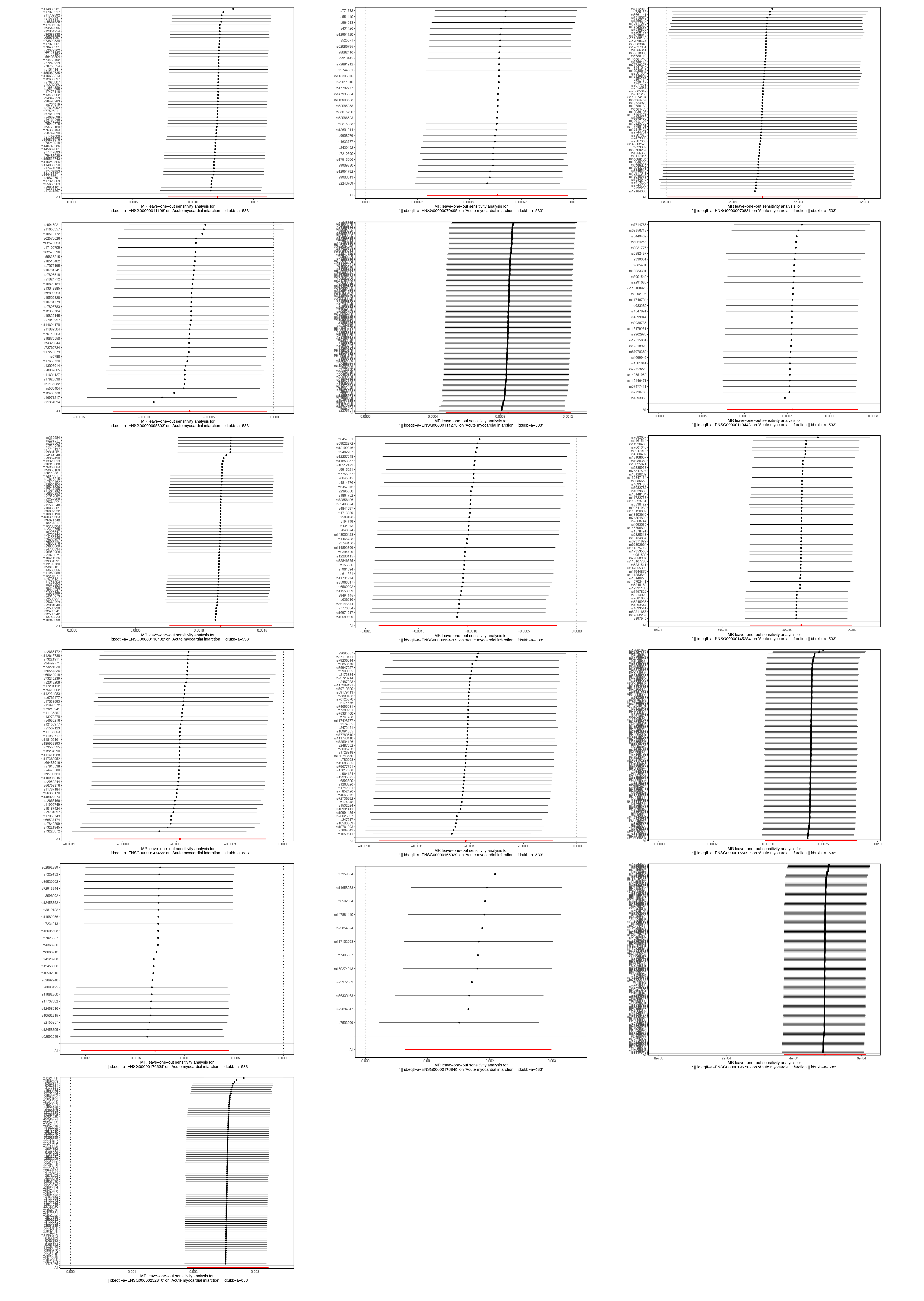

Supplement: Supplementary Figure 4 — Leave-one-out sensitivity analysis plot. This plot displays the overall effect estimate on AMI from the remaining SNPs after sequentially excluding each individual SNP. The results remained stable, indicating that the analysis was notdisproportionately driven by any single SNP. [file Image4.tif]
